# Epidemiology, management and outcomes of Graves’ disease—real life data

**DOI:** 10.1007/s12020-017-1306-5

**Published:** 2017-05-06

**Authors:** Y. S. Hussain, J. C. Hookham, A. Allahabadia, S. P. Balasubramanian

**Affiliations:** 1 Endocrine Surgery Unit, Directorate of General Surgery, Sheffield Teaching Hospitals NHS Foundation Trust, Sheffield, UK; 2 Directorate of Diabetes and Endocrinology, Sheffield Teaching Hospitals NHS Foundation Trust, Sheffield, UK; 3 Department of Oncology and Metabolism, University of Sheffield, Sheffield, UK

**Keywords:** Graves’ disease, Thyroid, Hyperthyroidism, Anti-thyroid drugs, Surgery, Radio-iodine

## Abstract

**Purpose:**

Treatment options in Graves’ disease are clearly defined, but management practices and the perceptions of success are varied. The outcomes of treatment in large consecutive cohorts of Graves’ disease have not been well characterised. The study describes the epidemiology, management strategies and medium term outcomes following anti-thyroid drug treatment, radio-iodine ablation and surgery in Graves’ disease.

**Methods:**

All patients (*n* = 659) who received treatment for a new diagnosis of Graves’ disease in secondary care over a 5 year period were included with a median (interquartile range) follow-up of 42.9 (29–57.5) months.

**Results:**

The age adjusted incidence of adult onset Graves’ disease in Sheffield, UK was 24.8 per 100,000 per year. Excluding 35 patients lost to follow-up, 93.1% (*n* = 581) were controlled on anti-thyroid drug treatment. Of these, 73.6% went into remission following withdrawal of anti-thyroid drugs; 5.2% were still undergoing initial therapy; 13.3% lost control whilst on anti-thyroid drugs; and 7.9% went on to have either surgery or radio-iodine ablation whilst controlled on anti-thyroid drugs. Of the 428 patients who achieved remission, 36.7% relapsed.

Of 144 patients who had radio-iodine ablation treatment, 5.6% relapsed and needed further treatment. Of 119 patients having surgery, 5.2% had long-term hypoparathyroidism and none had documented long-term recurrent laryngeal nerve palsy.

**Conclusions:**

In the follow-up, 39.9% of patients underwent surgery or radio-iodine ablation with little morbidity. Up to two-thirds of patients who achieved remission did not relapse. Data on effectiveness and risks of treatments for Graves’ disease presented in this study will help clinicians and patients in decision making.

## Introduction

Graves’ disease is the commonest cause of hyperthyroidism, typically presenting in patients between 40–60 years [1, 2]. Auto-antibodies, primarily Thyroid stimulating hormone Receptor Antibody (TRAb), are the driving force behind the disease but underlying mechanisms are still not completely understood. Good epidemiological data on the disease is lacking as epidemiological studies focus on ‘hyperthyroidism’ in general and not specifically on Graves’ disease [3].

Clinical and biochemical features of thyrotoxicosis in addition to an elevated TRAb, ophthalmopathy (and/or dermopathy), or diffuse increase on radio-active iodine or technetium uptake scan confirm a diagnosis of Graves’ disease [4]. Radio-isotope scan helps to differentiate between thyroiditis and other causes of hyperthyroidism [5]. Thyroid ultrasound can give information on vascularity, presence of thyroid nodules and size of the thyroid gland [6].

The approach to investigation and treatment of Graves’ disease varies widely [7]. In general, patients are started on thionamide medications to control thyroid hormone production and release, thereby relieving symptoms. Methimazole or carbimazole (which is converted to methimazole in the blood stream) is invariably used as first-line treatment. Due to the risk of hepatotoxicity, propylthiouracil is usually reserved for those who are intolerant to carbimazole, women in the first trimester of pregnancy, or those in thyroid storm [8–10]. The primary aim of anti-thyroid drug (ATD) treatment is to achieve normalisation of thyroid hormone production and induce remission of disease; occasionally it may be to prepare patients for radio-iodine ablation (RIA) or surgery [10]. One of two strategies is used—titration of dose to biochemical response, or block and replace treatment (i.e., fixed high dose ATDs given in combination with thyroid hormone replacement) [11]. A course of ATD treatment usually lasts for between 6–18 months as treatment beyond this has not been shown to have any additional benefits [11]. Some patients continue with low dose ATDs in the long term. This approach is also used in relapsed Graves’ disease as an alternative to RIA or thyroidectomy [12].

Relapse following ATD treatment is between 30–40% in the first 12 months and approximately 50–60% in long term [13, 14]. Following failure of medical therapy, definitive treatment with either surgery or RIA is considered. RIA is more popular in North America and is often used as a first-line therapy for Graves’ disease. However it is not suitable for pregnant patients and those with severe eye disease. Alternatively, thyroidectomy is effective in inducing remission, has a negligible relapse rate and is particularly useful in patients unsuitable for RIA and those with large goitres [1]. However there is potential for complications such as recurrent laryngeal nerve (RLN) damage and hypoparathyroidism. In the UK, there is significant variation in choice of definitive management for Graves’ disease. Worryingly, clinicians’ perceptions on the risks and benefits of each option vary widely [15].

Good quality data information on short and long-term outcomes of treatment in consecutive cohorts of patients with Graves’ disease is lacking. To our knowledge, there is no study describing natural history following initial diagnosis and outcomes from ATD, surgery and radio-iodine in a single cohort of patients. The aim of this study is to explore the epidemiology, clinical features and short to medium-term outcomes following management of Graves’ disease in a consecutive cohort of newly diagnosed patients managed in secondary care in the UK.

## Methods

This is a retrospective cohort study of patients treated for a new diagnosis of Graves’ disease at Sheffield Teaching Hospitals (STH) over a 5 year period (July 2008 to June 2013). Patients were identified by exploring a database (ERUS) of correspondence in the endocrine unit. Patients diagnosed during pregnancy and Graves’ disease or diffuse thyrotoxicosis secondary to drugs such as alemtuzumab and amiodarone are likely to be included in this cohort. Data on these patients such as demographics (age, gender and postcode), presentation, biochemical features (TSH - Thyroid Stimulating Hormone, FT4 - Free Thyroxine, FT3 - Free Tri-iodothyronine, TRAb - TSH Receptor Antibody and TPO - Thyroid Peroxidase), treatment, outcomes and status of disease when last available was collected from electronic records. Data on smoking was not available.

The reference ranges for normal values for the various laboratory investigations include 0–34 IU/ml for TPO, 0.27–4.2 mIU/L for TSH, 12–22 pmol/L for FT4 and 3.1–6.8 pmol/L for FT3. Results for TRAb are presented as either positive (>1.5 IU/L), indeterminate (1–1.5 IU/L) or negative (0–0.9 IU/L). The BRAHMS TRAK human LIA method was used for the TRAb assay. For patients where multiple results prior to treatment were available, the first available thyroid profile and auto-antibody results prior to starting medical therapy have been used for analysis. Where a date of starting medical therapy was not available, it was substituted by the date of diagnosis.

The outcome of initial medical treatment was determined from biochemical response (FT4 and TSH) and clinician’s interpretation of the biochemistry and recorded as follows:

- Controlled disease—normalisation of biochemistry whilst on ATDs or within 1 month of withdrawal of ATDs
- Disease remission—patients whose disease was controlled with ATDs and where control was maintained for at least a month after withdrawal of medical treatment
- Uncontrolled disease—persistently abnormal biochemistry despite ATDs or intolerant to ATDs
- Unknown—where data on response was not available

Patients that relapsed after initial remission with ATDs were started on ATDs again either as a further course of treatment or as a bridge to definitive treatment with surgery or RIA. In patients undergoing RIA therapy, goitre size was estimated by palpation and technetium scan to determine dose of radio-active iodine. In general, RIA therapy was avoided in patients with eye disease. However, when used in accordance to patient preference, prednisolone was started 2–3 days prior to treatment and gradually reduced over 3 weeks after treatment. RIA therapy and follow-up was provided as an outpatient treatment. In patients undergoing surgery, total-thyroidectomy or near-total thyroidectomy (TT) was performed. Pre-operative laryngoscopy, anaesthetic assessment and biochemical testing to ensure adequate control of thyroid levels were regularly performed. Lugol’s iodine (0.3 ml TDS for 10 days pre-operatively) was routinely used to reduce gland vascularity. Patients with inadequate control of hyperthyroidism were admitted for monitored in-patient treatment for 7–10 days prior to surgery. Treatment included a combination of ATD, Lugol’s iodine, beta-blockers and cholestyramine. Patients were typically discharged 1 day after surgery after initiation of thyroxine. Calcium levels were monitored after surgery and post-operative laryngoscopy was performed selectively in the initial part of the study (if patients had voice or swallowing problems) and routinely in the latter part of the study (as practice had changed following guidelines from the British Association of Endocrine and Thyroid Surgeons).

Patients were followed up for a median (IQR - interquartile range) time of 42.9 (29.0–57.5) months after the date of diagnosis. The date when information was last collected was July 2015.

All data was recorded on an Excel spreadsheet. A number of key variables were validated by an independent second observer. Data analysis was primarily performed using the IBM SPSS statistics version 21 software package; however, some calculations including incidence rates were done on Microsoft Excel 2007. For calculation of incidence rates, only patients from within the catchment area for STH were used. Population data was taken from the April 2011 census to calculate a crude incidence rate for adult Graves’ disease [16]. The number of people in Sheffield stratified by age and gender were obtained and the male to female ratio for the adult population was extrapolated from the ratio in the overall population. This was used to calculate age and gender adjusted incidence rates for each of the included 21 areas identified by the first part of the postcode. As some of the ‘S’ postcodes were in catchment areas for other nearby hospitals, all areas where the age and gender adjusted incidence rates were below 0.050 per thousand per year were excluded. This threshold was the level at which all relevant (i.e., STH) catchment areas were captured in the incidence calculations.

The Pearson *χ* ^2^ test with continuity correction or Fisher’s exact test was used to determine associations and differences in categorical data sets. The Mann–Whitney *U* test was used for comparisons of continuous data. A significance value of *P* < 0.05 was used in all cases. The Kaplan–Meier method was used to produce a survival curve comparing relapse rates between patients who had block and replace therapy and those who had titration therapy. To determine the influence of factors on the outcome of medical treatment, a binary logistic regression analyses was performed on variables shown to be significant on univariable analyses.

The project was approved as a service evaluation study by the clinical effectiveness unit in STH (Ref. 41213) and given the observational nature of the study, approval from the regional ethics committee or individual patient consent was not deemed necessary.

## Results

Six hundred and fifty-nine patients treated for a new diagnosis of Graves’ disease over 5 years were included. The median (IQR) age at diagnosis was 44 (33–56) years. The cohort included one ‘paediatric’ patient who was diagnosed at the age of 16. Females accounted for 79.8% (*n* = 526) of patients, giving a crude ratio of 4:1 (female to male). The adjusted sex ratio was 3.9:1 when standardised to the gender distribution in the local population (1.03:1). 93.8% (*n* = 618) were electively referred from primary care and 6.2% (*n* = 41) presented to accident and emergency services.

**Fig. 1:**
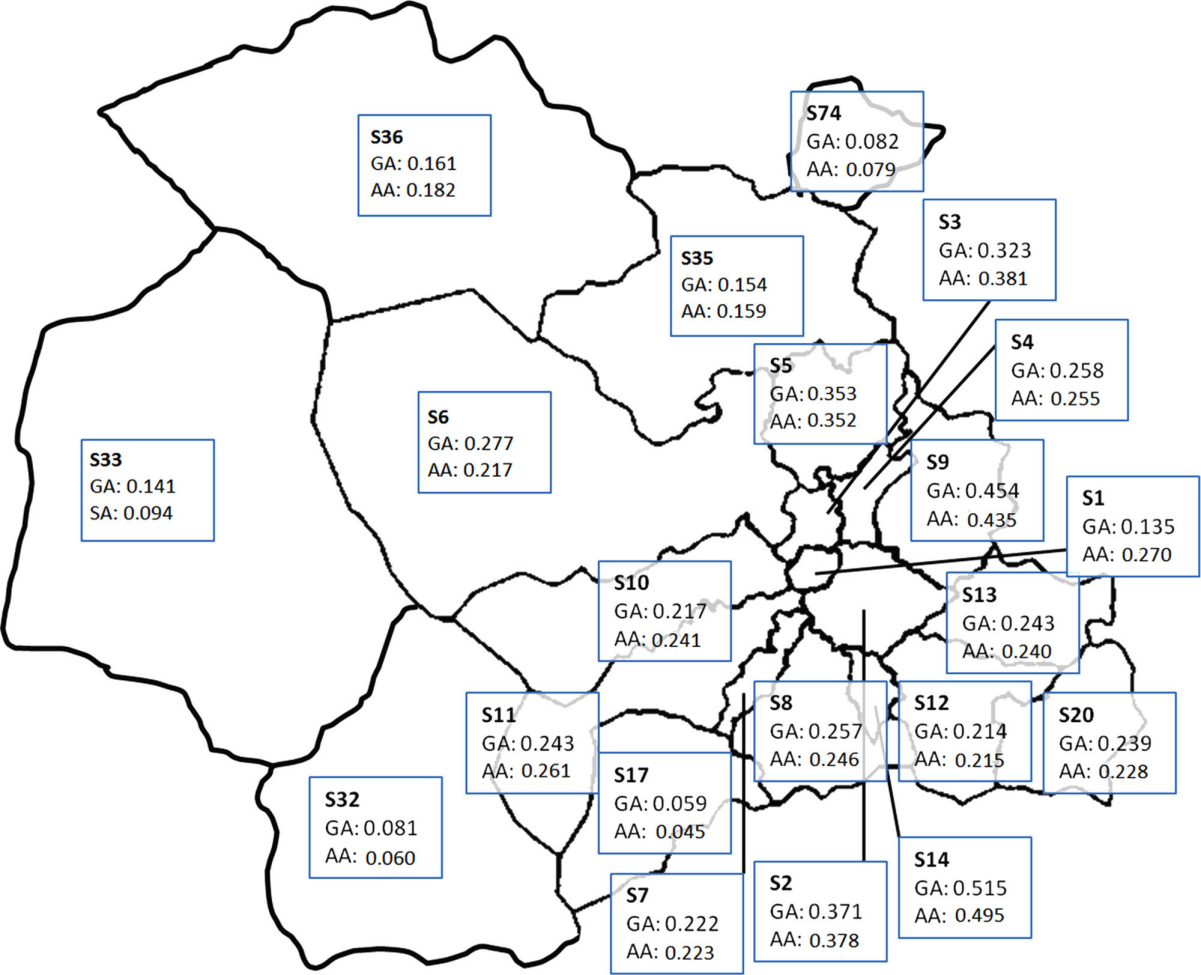
Postcode map of Sheffield (United Kingdom) with gender-adjusted and age-adjusted incidence rates for Graves’ disease. AA, age-adjusted incidence; GA, gender-adjusted incidence. Each outlined area refers to a postcode area in the city of Sheffield

Of the 658 adult patients identified, 644 lived in the Sheffield area (denoted by an ‘S’ postcode). After exclusion of patients in ‘S’ postcodes that were in catchment areas for other hospitals, 609 patients were included in incidence calculations. The adult population in the relevant postcodes was 490,555; as per the 2011 census. The overall crude incidence of Graves’ disease was 24.8 per 100,000 population per year. The age and gender adjusted incidence rates of adult Graves’ disease presenting to hospital are 24.9 and 24.8 per 100,000 per year, respectively (Fig. 1). Gender specific incidence of adult Graves’ disease was 11.0 and 37.9 per 100,000 population per year for males and females respectively.

**Fig. 2:**
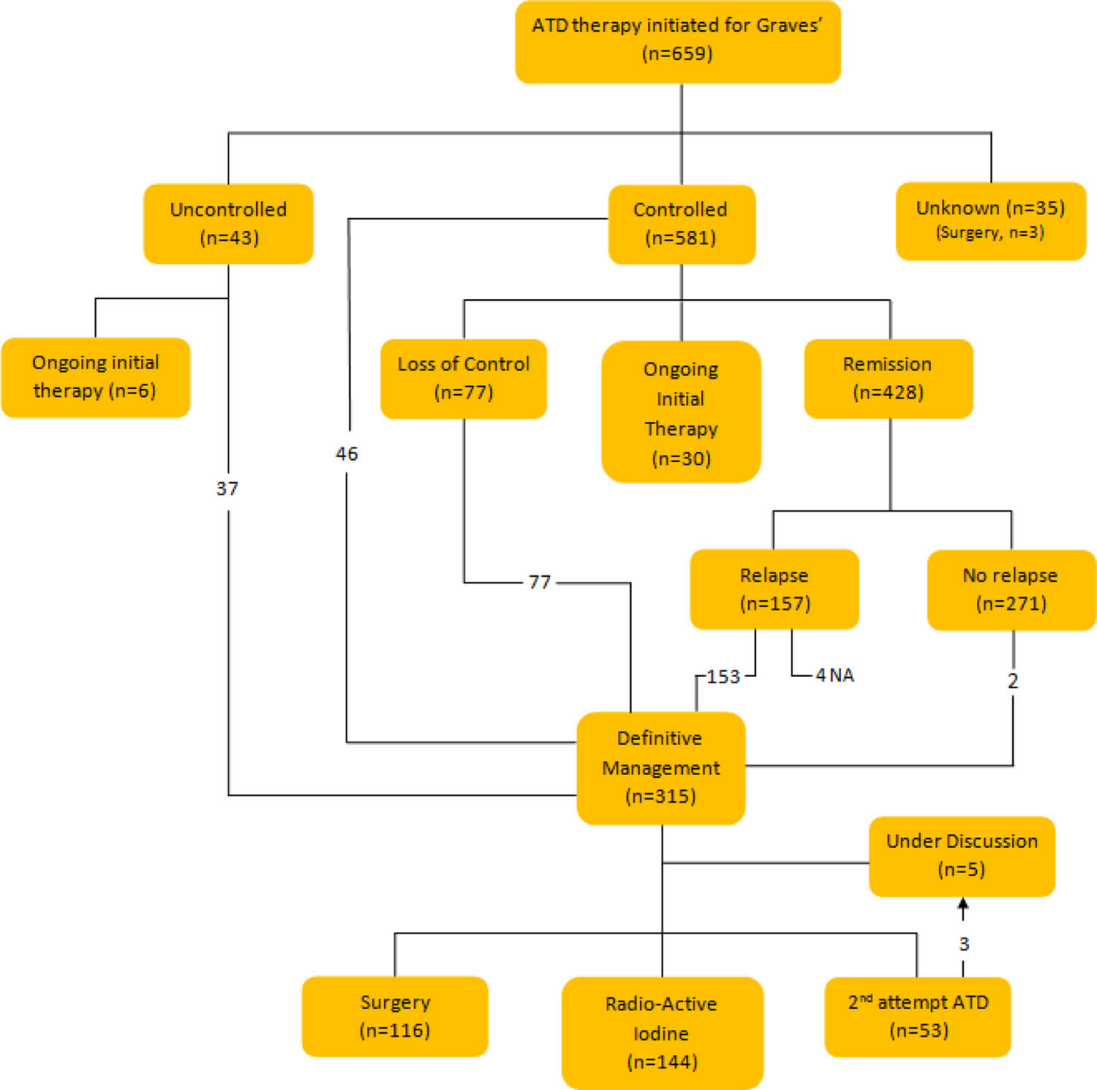
Flow chart depicting management pathway for patients with Graves’ disease

### Presentation and diagnosis

Of 372 patients where TRAb antibody results prior to initiation of medical therapy were available, 5.9% (*n* = 22) of patients had a negative TRAb, 2.4% (*n* = 9) were indeterminate and 91.7% (*n* = 341) were positive. A detailed comparison between TRAb positive and ‘indeterminate or negative’ groups is given in Table 1. TPO antibody levels prior to initiation of medical therapy were available in 323 patients. It was positive ( ≥ 35 IU/L) in 73.1% (*n* = 236) and negative (<35 IU/L) in 26.9% (*n* = 87).

**Table 1:** Comparison of TRAb positive and TRAb negative patients with Graves’ disease

	TRAb Positive (*n* = 341)	TRAb Indeterminate or Negative (*n* = 31)	*P* value
Median (IQR) age at diagnosis	46 (33–57)	43 (26–65)	0.99*
F:M ratio	3.8:1	6.8:1	0.357**
TPO positive	74.5% (*n* = 187)	50.0% (*n* = 12)	0.016**
Median FT4 (IQR) prior to treatment (pmol/L)	32.8 (24.4–44.5)	28.0 (24.5–35.2)	0.02*
Median FT3 (IQR) prior to treatment (pmol/L)	13.3 (8.9–19.5)	8.2 (6.9–11.4)	<0.01*
Technetium scan done?	25.2% (*n* = 86)	12.9% (*n* = 4)	0.089**
Outcome of medical treatment			0.004**
Uncontrolled or loss of control	64 (19.9%)	2 (6.5%)	
Controlled or remission	169 (52.5%)	27 (87.1%)	
Relapse	89 (27.6%)	2 (6.5%)	
%needing RIA / surgery	39.6%	9.7%	0.522**

*Mann–Whitney *U* test

**χ ^2^

All patients except one had a suppressed TSH prior to treatment initiation. One patient with TSH in normal range had clinical features of hyperthyroidism, raised FT3 and FT4 levels, positive TRAb, strong family history of thyroid disease and a medical history of autoimmune disease. It was felt that despite normal TSH levels, this patient would benefit from therapy. The median (IQR) FT4 and FT3 levels prior to treatment were 33.0 (24.6–47.0) pmol/L and 12.9 (8.8–20.4) pmol/L, respectively.

Technetium (^99m^Tc) uptake scan was performed in only 26.7% (*n* = 176) of patients. In 81.8% (*n* = 144) of these patients it was performed in preparation for RIA therapy. Ultrasound scan was recorded in 67 patients and in 19 of these prior to treatment initiation.

### Management

Primary care physicians initiated ATD treatment in 38.9% of patients (246/633), where data was available. Carbimazole was the preferred thionamide, used in 98.2% (*n* = 647). Carbimazole was used alone in 88.0% (580/659) as opposed to PTU in 1.8% (10/659). Both thionamides were used sequentially in 10.2% (67/659) and in 2 patients carbimazole was started after methimazole or thiamazole, both of which were started abroad. A block and replace regime was used in 83.0% (*n* = 547) of patients. For patients where the duration of treatment was available (*n* = 451), the median (range) duration of treatment for the ‘block and replace’ and ‘titration’ regimes were 8 (3–36) and 16 (0–24) months, respectively. The commonest starting dose of carbimazole was 40 mg once daily, which was used in 71.9% (402/559) of cases where starting dose was available.

Figure 2 summarises the flow of patients through their treatments and their outcomes.

**Fig. 3:**
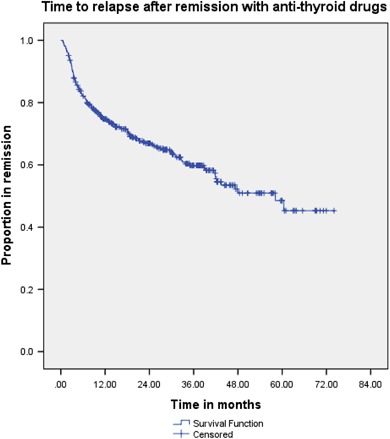
Kaplan–Meier survival curve showing time to relapse in patients who were considered to have remission after ATD treatment

Excluding patients lost to follow-up (*n* = 35), 93.1% (*n* = 581) were controlled on initial ATD treatment. In patients where control was not achieved (6.9%, *n* = 43), 14.0% (*n* = 6) were continuing initial therapy at last follow-up, 41.9% (*n* = 18) underwent surgery and 44.2% (*n* = 19) had RIA therapy. In the controlled group (*n* = 581);

- 7.9% (*n* = 46) had definitive management [surgery (*n* = 18) or RIA therapy (*n* = 27)] for a variety of reasons including co-existing goitre, side effects to treatment, severe disease and patient preference.
- 13.3% (*n* = 77) suffered a loss of control whilst on ATDs and of these 44.2% (*n* = 34) had surgery, 36.4% (*n* = 28) had RIA therapy and 19.5% (*n* = 15) had further ATD therapy.
- 5.2% (*n* = 30) were still controlled and on initial therapy at last follow-up (this includes both those on long-term ATDs and those in whom the goal was to achieve remission).
- 73.7% (*n* = 428) of the controlled group were able to achieve remission after withdrawal of ATD therapy. Of those who achieved remission (*n* = 428), 36.7 % (*n* = 157) had relapsed at last follow-up.

A number of factors were evaluated for their potential to predict failure of medical therapy (defined as patients uncontrolled on medical treatment, patients that lost control while on treatment and patients that relapsed after cessation of medical treatment) and therefore the need for definitive treatment. Failure of medical therapy was significantly higher in the block and replace group vs. the titration group (46.8 vs. 29.9%, respectively, *p* = 0.003, *χ* ^2^ test with continuity correction) and significantly lower in TRAb-negative compared to TRAb-positive patients (12.9 vs. 47.5% respectively, *p* < 0.001, *χ* ^2^ test with continuity correction). Similarly, patients who failed medical therapy had significantly higher FT4 and FT3 levels before treatment (*p* < 0.001, Mann–Whitney *U* test). Age at diagnosis (*p* = 0.113; Mann–Whitney *U* test), gender (*p* = 0.856; *χ* ^2^ with continuity correction) and TPO levels (*p* = 0.654; *χ* ^2^ test with continuity correction) did not have a significant impact on the risk of failure of medical treatment. Binary logistic regression analyses including significant variables (TRAb status, FT4 and FT3 levels and type of treatment) showed that only FT3 levels (*p* = 0.015) and TRAb (*p* = 0.005) status predicted failure of treatment.

In the patients who had remission after initial ATD treatment, six patients had less than six weeks of treatment and may be considered to have gone in to ‘spontaneous’ remission. In this group TRAb was positive in 4, equivocal in 1 and negative in 1. TPO was positive in 5 patients and no result was available for the remaining patient. None of these 6 patients relapsed. In the group who did not relapse (*n* = 271), two patients had surgery—one for a co-existing indeterminate thyroid nodule and the other for a prominent goitre.

Figure 3 shows a survival curve demonstrating time to relapse in patients that had remission after anti-thyroid drug treatment. In the group that relapsed (*n* = 157), 28.0% (*n* = 44) had surgery, 44.6% (*n* = 70) had RIA therapy and 24.2% (*n* = 38) had a second round of ATDs. Of the remaining 5 patients, one died shortly after relapse (of other causes), one was lost to follow-up, two developed hypothyroidism subsequently and one was considering definitive management at last follow-up.

Table 2 compares demographic and clinical characteristics of patients who had surgery and RIA as definitive treatment for Graves’ disease. Surgery was performed more often in female patients as compared to males. Age, FT3 and FT4 levels prior to treatment were also found to be significantly associated with the type of definitive management; surgery being favoured in younger patients and those with more severe disease.

**Table 2:** Comparison of demographic and clinical features of patients undergoing surgery and RIA therap**y**

	RIA therapy (*n* = 144)	Surgery (*n* = 116)	*P* value
Median (IQR) age at diagnosis	50.5 (43.0–65.3)	43.5 (30.1–51.0)	<0.001**
Females/males (incidence 3.9:1)	2.5:1	6.4:1	0.003*
Mean FT4 level at presentation	36.4 (26.1–49.3)	43.6 (31.9–54.3)	0.037**
Mean FT3 level at presentation	13.8 (9.7–20.8)	20.9 (13.6–28.2)	<0.001**
Median (range) age at intervention	50.9 (18.7–83.2)	39.5 (19.0–66.5)	<0.001**
Positive TPO status before treatment	79.3% (*n* = 58)	72.9% (*n* = 59)	0.404*
Positive TRAb status before treatment	97.3% (*n* = 73)	98.5% (*n* = 65)	1.000*
Indications for definitive treatment	Uncontrolled on ATD 13.2% (*n* = 19)	Uncontrolled on ATD 15.5% (*n* = 18)	
	Loss of control on ATD—19.4% (*n* = 28)	Loss of control on ATD—29.3% (*n* = 34)	
	Relapse—48.6% (*n* = 70)	Relapse—37.9% (*n* = 44)	
	Others—18.8% (*n* = 27)	Others—17.2% (*n* = 20)	

*Pearson-*χ* ^2^

**Mann–Whitney *U* Test

One hundred and fourty-four patients had RIA therapy for Graves’ disease. The median (range) dose of activity for radio-iodine treatment was 516 MBq (374–934). The median (IQR) follow-up time after RIA treatment was 25.7 (12.0–47.1) months. Of the patients who had RIA therapy and a minimum of 3 months follow-up (*n* = 135), relapse was observed in 8 patients (5.9%); all occurring within 6 months of RIA therapy. Four of these went on to have further RIA therapy and the remaining four were managed with ATDs.

One hundred and nineteen patients had surgery. Of 115 patients where histology was available, 5 (4.4%) had malignant histology; all these tumours were papillary subtype of differentiated thyroid cancer. Post-operative complications studied included hypocalcaemia and RLN injury. Calcium results were available in 97.5% (*n* = 116) of patients. One patient had pre-operative hypocalcaemia and was excluded. 68.1% (*n* = 79) had post-operative adjusted calcium levels of over 2.1 mmol/L. 16.4% (*n* = 19) had transient hypocalcaemia which resolved without treatment; 10.3% (*n* = 12) had hypocalcaemia needing treatment and resolved in 6 months or less and 5.2% (*n* = 6) needed treatment for hypocalcaemia lasting for over 6 months. Data on post-operative laryngoscopy was available in 73 of the 119 patients. Of these, 94.5% (*n* = 69) were normal and 5.5% (*n* = 4) had a transient vocal cord palsy (lasting less than 6 months). No patient had long-term vocal cord palsy. If the group of patients in whom data on post-operative laryngoscopy was not available (*n* = 46) are assumed to have had no RLN injury, the percentage of patients in whom there is no injury increases to 96.1% (*n* = 98).

## Discussion

This study is the first to report on the incidence of Graves’ disease in a UK population and examine the outcomes of a consecutive cohort of patients who have had ATDs, radio-iodine and surgery.

The incidence of Graves’ disease was 24.8 cases per 100,000 with an adjusted female to male ratio of 3.9:1. Studies have described incidence of thyroid disorders [17, 18] but studies specifically on Graves’ disease are rare [3]. In Sweden, the reported incidence of Graves’ disease (2003–2005) was 21.4 per 100,000 per year with a F:M ratio of 5.6:1 [19]. The ratio of 3.9:1 reported in this study is however in keeping with other studies in Iceland and Sweden that reported a gender ratio of 4:1 in hyperthyroidism in general [3]. The true incidence of hyperthyroidism may vary between populations and factors such as iodine levels in drinking water are thought to have a significant impact [20]. However, comparisons between populations are difficult due to different inclusion criteria, TRAb methods and diagnostic methods [17, 21].

TRAb had a 91.7% sensitivity for Graves’ disease in our cohort; similar to previous literature where TRAb has been negative in up to 10.0% [1, 21]. It has been argued that TRAb negative patients may not have Graves’ disease; but usually, a combination of clinical features, biochemistry, antibody profile and imaging are used to make the diagnosis and this combination remains the gold standard [21]. UK guidelines currently recommend that TRAb testing is not required to determine the cause of hyperthyroidism, particularly if the clinical features are suggestive of Graves’ disease [22]. TPO antibody was positive in 73.1% of those tested which is in keeping with a similar previous studies [23, 24].

Technetium (^99m^Tc) uptake scan is not routinely performed and is used less frequently since the introduction of more accurate TRAb assays [6]. It is performed in preparation for RIA therapy to assess gland size and calculate dose of radio-iodine for ablation. 81.8% of patients who had a Technetium (^99m^Tc) uptake scan had RIA therapy in this cohort, suggesting that the scan was used primarily in preparation for RIA treatment.

In this series, the rate of initial control with ATDs was 93.1%. The overall failure rate of first-line treatment with ATDs (those with uncontrolled, loss of control or relapsed disease) was 60% (271/452) at last follow-up. This excludes patients with unknown outcomes (*n* = 35), those with ongoing therapy (*n* = 36) and those who had definitive management despite control (*n* = 46). Other studies have estimated similar failure rates of around 50–60% [13, 14, 25]. Reports do not clearly distinguish between patients who fail treatment early in the course and those who relapse after remission. In this study, of those who went into remission and where data on follow-up times were available, relapse occurred in 25 and 33% at 12 and 24 months, respectively.

Carbimazole was the preferred thionamide, used in 98.2% of patients. Block and replace regimes were used in 83% of patients and these patients failed medical therapy more often than those who had titration regimes (46.8 vs. 29.9%, respectively). But, the superiority of one treatment over the other remains unclear [26]. Nedrebø et al. [27] showed no significant difference in remission rates between patients treated with block and replace vs. titrate after 24 months follow-up in a prospective cohort of 218 patients. Grebe et al. [28] showed similar findings using higher doses of carbimazole for block and replace (100 mg) although this group had significantly more side effects. It has been argued that titration regimes are favourable due to reduced side effect profile [29–31] and the independence of remission rates from drug type and dose [31, 32]. A recent observational study of 450 patients also found no evidence to support the hypothesis that block and replace regimes resulted in more stable thyroid function [24]. Our results may be explained in part by the fact that regimes were of varying lengths unlike in most prospective, comparative studies. The titration group were treated on average for twice the length of time compared to block and replace group (16 vs. 8 months, respectively).

Identification of predictors of relapse in Graves’ disease would help tailor treatment to specific patient groups. Various factors such as smoking status, TRAb levels before and after treatment, low TSH, goitre size and more recently, stress events, are suggested to be significant [25, 27, 33–39]. We found that age, gender and TPO status did not influence failure, confirming previous findings [27, 35, 36, 40]. We found that patients with more severe disease (higher FT3 and FT4) were significantly more likely to fail medical treatment. This is contrary to findings of Glinoer et al. [37], where both total-T3 and free-T3 and T4 did not predict relapse. However, they were treated with an initial block and replace followed by titration regime which is different to our study. A significant number of patients in this study also continued to take exogenous thyroxine during the 12 month follow-up period. Cappelli et al. found in a prospective study of 216 patients that patients who did not relapse after first round of ATDs had a significantly lower T4 than those that did relapse [38].

Our results indicate that patients that were TRAb positive are significantly more likely to fail medical treatment, confirming findings in other studies [33–35]. Quadbeck et al. [40] demonstrated that TRAb was a predictive marker for relapse in Graves’ disease, but was dependent on TSH levels. Hoermann et al. also reported similar findings [36]. However, Scott et al. [33] concluded that at high levels, TRAb alone was an independent predictor of relapse.

We found that surgery was, significantly, more commonly performed in younger, female patients with more severe disease at presentation (higher FT3 and FT4). This may be because some exclusion criteria for RIA (such as pregnancy and breast feeding) apply exclusively to females. It is also expected that younger patients will be more suitable for surgery with fewer co-morbidities. TPO and TRAb status at presentation did not predict choice of definitive treatment.

In patients with a minimum of 3 months follow-up post-RIA therapy (*n* = 135), relapse rate was 5.9%; all occurring within 6 months. This is in keeping with previous studies which have reported success of RIA therapy to be over 85% [41, 42].

In surgical patients, 5.5% had temporary RLN damage; transient and long term post-operative hypocalcaemia occurred in 31.9 and 5.2%, respectively; and incidental papillary thyroid cancer rate was 4.4%. The complication rates were higher than those reported in a prospective study in Poland (transient rate of 25%, long-term rate of 0%) on including 96 patients who underwent total thyroidectomy [43] However, the definitions of transient and long-term hypoparathyroidism differ greatly between centres and this can significantly influence rates [44]. There is a wide range (5.4–19%) in the rates of incidental thyroid cancer following thyroidectomy reported in the literature [43, 45–47]. This variation may be due to differences in the underlying cohorts, the approach to diagnosis and the criteria for considering surgical treatment; especially in patients with thyroid nodules where the incidence of thyroid cancer has been reported to be much higher [45, 47]. Stahopoulos et al. [48] similarly found long-term hypoparathyroidism to be 5.4% post-operatively with no patients having permanent RLN damage. A recent study from our unit [49] on post-thyroidectomy hypocalcaemia reported transient and long-term rates of 29.0 and 5.5% in patients undergoing total thyroidectomy (for all indications) over a 3.5 year period. No patients had recurrence of hyperthyroidism following surgery, consistent with other recent literature [48, 50]. In conclusion, surgery is an effective and safe management option for Graves’ disease.

Currently there is no consensus on the optimal management strategy after failure of ATDs. The choice between RIA, thyroidectomy and a second round of ATDs appears to be influenced by physician, patient, institutional and geographical preferences. There may be some reluctance amongst clinicians to recommend thyroidectomy unless RIA and ATDs are contraindicated but preference for thyroidectomy appears to be increasing [48, 51, 52]. An analysis by Haejin et al. [53] found that total thyroidectomy was the most cost-effective strategy following failure of first-line ATDs for the model Graves’ patient in the US. The choice of definitive management should be made on a case-by-case basis and our results will aid clinicians in choosing the best definitive treatment strategy for their patients following failure of ATDs.

This study is limited by its retrospective nature and the lack of a complete dataset. Incidence rates may have been under estimated as only patients presenting to secondary care were included. However, current practice in the UK is for all patients with hyperthyroidism to be referred to secondary care by their general practitioner. The comparisons between surgery and radio-iodine groups are subject to selection bias. Although the choice of treatment was based on recognised principles of management, the use of different treatments were subject to the treating clinician’s discretion and patient choice; resulting in significant heterogeneity. Data on the rationale for the use of various treatments was not recorded and detailed analyses of patients undergoing a second round of ATDs was not performed due to logistic difficulties and data availability. However, the data provides significant insight into practice in secondary care in a large UK hospital and a pragmatic understanding of the outcomes of treatment in real life.

In summary, this study has described the epidemiology of Graves’s disease in a western population. The short to medium-term outcomes following the management strategies employed will help clinicians and patients in decision making. Surgery and radio-iodine are both effective and associated with minimal morbidity but controlled trials are needed to evaluate their relative effectiveness in comparison to each other and to further treatment with ATDs.
